# Non-Contrast Radiation-Free NIR Lung Imaging

**DOI:** 10.3390/biomedicines13112757

**Published:** 2025-11-11

**Authors:** Jiří Votruba, Martin Drahanský, Tomáš Goldmann, Tomáš Brůha, Radim Kolář

**Affiliations:** 1 I. Clinic of Tuberculosis and Respiratory Diseases, General University Hospital in Prague, 128 08 Prague, Czech Republic; bruha@enex.cz; 2 Department of Criminalistics, Faculty of Security and Law, Police Academy of the Czech Republic in Prague, 143 00 Prague, Czech Republic; 3 Faculty of Information Technology, Brno University of Technology, 612 00 Brno, Czech Republic; goldmann@vut.cz; 4 Faculty of Electrical Engineering and Communication, Brno University of Technology, 616 00 Brno, Czech Republic; kolarr@vut.cz

**Keywords:** solitary pulmonary nodule, near-infrared imaging, translumination, bronchoscopy, pleuroscopy, image processing

## Abstract

**Background/Objectives**: Early localization of solitary pulmonary nodules (SPNs) remains challenging despite technological advances in endoscopic navigation, as the procedure often necessitates multiple ionizing imaging examinations. This study aimed to develop and evaluate a radiation-free optical method for SPN localization based on near-infrared (NIR) translumination. **Methods**: A miniaturized NIR light source was introduced into the bronchial tree to illuminate the lung parenchyma. The transmitted and scattered NIR light was detected in real time from the pleural side using minipleuroscopy and a CMOS camera. The approach exploits intrinsic differences in optical absorption and scattering between normal and pathological lung tissue, allowing visualization of the parenchymal micro-architecture without exogenous contrast agents. **Results**: In ex vivo porcine lungs, tissue structures were clearly visualized through up to approximately 4 cm of parenchyma. In a ventilated pig (*n* = 1), bronchial NIR illumination was consistently detected from the pleural cavity and produced distinct images of lobular structures and the bronchial mucosa. **Conclusions**: These feasibility findings demonstrate that NIR translumination can provide radiation-free intra-thoracic visualization and may serve as a valuable adjunct for biopsy guidance. Further quantitative validation and clinical translation are warranted to establish its applicability in human pulmonary procedures.

## 1. Introduction

*Lung cancer* still accounts for 12.3% of all new cases of cancer diagnoses [1,2]. Smoking is a well-documented cause of lung cancer, having been directly linked to 90% of cases. Lung cancers most commonly arise from the epithelial lining of the bronchial airways. Four histological types of lung cancer have been defined: small cell carcinoma, squamous cell carcinoma, adenocarcinoma, and large cell carcinoma. The latter three are often grouped into what is usually referred to as a non-small-cell carcinoma, accounting for approximately 85% of all lung cancers. If the disease is detected at a late stage, which is unfortunately the most common situation, the five-year mortality rate for patients is about 85%. An explanation for this poor survival rate is likely to be in the staging of the disease; at the time of diagnosis, only 20% of patients have localized disease. Even when the tumor is radiologically localized, the five-year survival rate is still only 30–40%. However, if found at the early stage of disease (usually presenting as a solitary pulmonary nodule on CT), the five-year survival is drastically improved, reaching 80% in developed countries. This is why lung cancer screening was introduced 7 years ago, resulting in a steep increase in the number of solitary pulmonary nodules (SPNs) caught during low-dose spiral chest CT screening.

*Lung cancer screening* has increased the detection of small SPNs, but reliable localization for minimally invasive diagnosis remains difficult and often requires multiple CT-guided or navigation-assisted procedures, each with costs, radiation, and complication risks. Near-infrared (NIR) optical methods provide complementary contrast based on intrinsic tissue scattering/absorption and can be implemented with compact hardware. While NIR fluorescence with indocyanine green (ICG) is increasingly used for thoracic surgery and nodule localization, it requires systemic dye administration and carries dye-related limitations. Here, we explore a contrast-free NIR translumination strategy that places the light source inside the airway and detects transmitted/scattered photons from the pleural side to reveal the parenchymal structure in real time.

The Nelson study conducted in Europe achieved results consistent with and even exceeding those of the National Lung Screening Trial [3] (performed by the National Cancer Institute, USA) [4], although the methodology of this study was in some ways different. It is therefore certain that many early lung tumors will be (and are already) diagnosed as *solitary pulmonary nodules*. Thus, the current effort in pneumology diagnostics is focused on solitary pulmonary nodules. Nowadays, a diagnosis of lung cancer is usually based on tissue diagnosis through *endoscopic* or *transparietal approaches*. As the diagnosis will move from advanced stages, where morphological evidence is usually not difficult to find, to earlier stages, new requirements for the diagnosis of small solitary pulmonary nodules will arise.

However, in addition to clear positive results, screening also brings a number of problems. The most significant is the vast number of false positives found in LDCT [5], forming the so-called over-diagnosis effect in this population. This systematic screening error leads to unnecessary treatment and the associated morbidity and mortality for findings that would not have otherwise reduced the patient’s quality of life. In the NLST, over-diagnosis occurred 19% of the time [6]. This issue is most easily expressed by the following simple enumeration. For each cancer death prevented by an early diagnosis of lung cancer, we would create 320 cases of “over-diagnosis”. Hence, futile investigation in these cases creates large-scale ethical, economic, and medical problems. We are therefore exploring new methods of narrowing down the screening group and evaluating CT images to reduce unwanted screening effects. The most promising pretests are analyses of serum or exhaled air and/or spirometric examination. *Chronic obstructive pulmonary disease* is associated with an increased risk of developing lung cancer. In men, a forced expiratory volume in 1 s (FEV1) of less than 70% is associated with a 2.23-fold increased risk, and for women, it is increased 3.94-fold [7]. Other methods that have been proposed to narrow down the screening population are based on molecular biology, but these have been tested in the setting of lung cancer recurrence only [8,9,10].

The selection of an optimal screening interval, which can be obtained in particular by processing the Nelson study’s data, is important. It appears that prolongation of the screening interval (in the Nelson study, it was 1 year, 2 years, and 2.5 years) brings a stationary incidence of diagnosed tumors to the screening round (0.8%) but worsens the average stage of newly diagnosed tumors in the last round of screening (i.e., 2.5 years) [11]. Thus, the 2.5-year interval is clearly too long, and screening should be kept at the NLST-recommended 12-month interval between screening rounds [12]. Another problem is that most of the research is focused on lung nodules that are detected at the beginning of screening. New nodules have been reported in different studies, although their significance has been quite varied. The ELCAP, I-ELCAP, PLuSS, and Mayo trials have shown different annual incidences of new nodules, as shown in [13,14]. These new nodules are typically fast-growing, and the available data shows that the probability of malignancy in such a newly emerging nodule is 1.6–7.5% [13,14]. Therefore, it may be necessary to choose a lower volume threshold for these new nodules than that for the first round of screening. The most cited models that aim to distinguish between malignant and benign nodules are the model of McWilliams and the American College of Radiology model (the Lung-RADS model published in 2014 [15]). The Lung-RADS model is used in virtually all American screening centers. It includes five categories that determine management based on the type of lesion and its size. Therefore, proper selection of patients seems to be most important for screening, and new screening programs should always include 3D image analysis. Multidisciplinary teams are necessary to make the right decisions about individual cases [16,17].

A solitary pulmonary nodule is a pathology up to 3 cm in diameter that is surrounded by normal lung tissue and is not the result of any other pneumopathy [18]. There are essentially three diagnostic possibilities in this case [19,20]. The first option involves an immediate referral for chest surgery. Although this approach carries a non-negligible risk of morbidity and mortality, it is also costly and should therefore be reserved for exceptional cases in which the diagnosis of malignancy remains uncertain. Another possible option is a *transparietal lung biopsy guided by CT or ultrasound* [21]. In addition to the radiation risk [22] for both the patient and the doctor, there is also a 40% risk of the patient developing pneumothorax, which is a potentially very serious complication in this group of fragile patients, with frequent emphysematous lung remodeling. Therefore, we only resort to this approach after repeated failure of endoscopic diagnosis. Until recently, *pulmonary endoscopy* alone had very limited value in the diagnosis of solitary pulmonary nodules. For morphological diagnosis, only 30 to 40% sensitivity has been reported. Combined with one of the new navigation methods, the success rate of endoscopic diagnosis for nodules of 2 cm or more in diameter can rise up to 85%, but smaller nodules are still a serious diagnostic problem, as they are often not associated with the bronchial lumen, and therefore, endoscopic diagnosis is difficult, if not impossible. New methods are therefore being developed, mainly based on electromagnetic or software navigation, tunnelization to the lesion, or ultra-thin bronchoscopy [23,24]. However, none of these methods have repeatable results, and therefore, an analysis of diagnostic efficiency is difficult (dependent on the technology, on the location of the lesion, and on the quality of the diagnostics). Yet navigation in lung endoscopy, along with the development of robotic endoscopy, is a very rapidly developing technological field.

*Raman spectroscopy* has become very popular recently. It has been shown that adding Raman spectroscopy to current white light bronchoscopy/autofluorescence bronchoscopy (WLB/AFB) improves its sensitivity and specificity for the detection of preneoplastic lesions in vivo [25,26]. All three modalities were combined and demonstrated in vivo detection of preneoplastic lesions in 26 patients. The authors were able to obtain a sensitivity of 96% and a specificity of 91%. Continued work is, however, needed to develop an optimized probe and data analysis model for this trimodal application. Additional work is being undertaken to identify disease-specific biomarkers from Raman spectra which correlate with biochemical signatures of specific histological signs of malignancy. As for solitary pulmonary nodule detection, Raman spectroscopy has been shown to be useful as a device for monitoring nodules. A special optical catheter Raman probe can be directed to the site of a suspected SPN by the means of fluoroscopy, which can confirm different tissue compositions prior to biopsy [27,28,29].

Recently, near-infrared (NIR) image-guided surgery has been proposed for identifying nodules in solid organs [30,31,32,33]. This technique is based on the application of a specific fluorescent dye (typically indocyanine green), followed by imaging with an NIR camera [34,35,36]. With this technique, thoracic surgeons were able to visualize 16 of 18 small SPNs in the peripheral area of the lungs during videothoracoscopy [30,37,38]. The recent advancements in this technique have led to NIR-II imaging at wavelengths above 1000 nm [39] with the application of nanoparticles and quantum dots as a contrast agent. In spite of the high visual contrast of these pathological tissues, contraindications (renal dysfunction, iodine hypersensitivity, uremia) are a limiting factor of these NIR contrast techniques [40,41,42].

Compared with white light endoscopy, NIR wavelengths reduce light absorption and scattering, potentially improving penetration and the signal-to-background ratio. Recent NIR-II developments (1000–1700 nm) have further motivated optical approaches to deep tissue imaging; however, a simple, dye-free solution for intra-thoracic visualization has not been demonstrated clinically. We therefore evaluated its feasibility in porcine tissue and an initial in vivo experiment.

## 2. Materials and Methods

Our aim is to develop a diagnostic infrared imaging kit for morphological SPN detection using real-time visualization. This principle is based on the use of near-infrared light to make solitary pulmonary nodules visible. The method of lung tissue illumination and the acquisition of video sequence data obtained from the lungs, in combination with the in vivo implementation, are completely unique and have not yet been published.

It is well known that transmittance, which is the ratio between the transmitted radiant flux and radiant flux that has reached the surface of matter, depends on the optical characteristics of the matter being investigated. This is represented by the absorption coefficient $\mu_{a}^{\lambda }$, the scattering coefficient $\mu_{s}^{\lambda }$, and the index of refraction *g*, as well as the anisotropy, with respect to the isotropic properties of the matter. With respect to the tissue being examined, it can be assumed that the mentioned optical characteristics are in strong correlation with the morphological characteristics of the tissue. This is because scattering arises due to the relative refractive index mismatching at the boundaries between two such media or structures, for instance, between the extracellular fluid and the cell membrane, which is represented by inhomogeneities. It is also essential to note that cell density also plays an important role in the outcome of scattering; for example, tumor clusters are characterized by a different density than that of normal tissue. Light scattering and absorption can provide information both about the tissue structure and the chromophore content, and these are features that can be used to distinguish between normal tissues, malignant lesions, and other pathologies.

It follows that NIR light penetrating normal lung parenchyma will undergo different attenuation than the light penetrating through pathological tissue, based on the tissue properties mentioned above, and therefore gives us information about its morphological properties and the presence of SPNs as well.

The possibility of direct visualization of the *solitary pulmonary nodules* through *translumination* with different light sources has not yet been described or investigated in the area of the lung parenchyma. The indisputable advantage of such an approach potentially becoming included into routine clinical pleuroscopy would be the possibility of real-time tissue biopsy under direct visual control, together with SPN treatment, in one session. Since clinical pleuroscopy is an examination that has practically no contraindications, except for patient non-compliance and in individuals with severe respiratory failure or severe coagulation disorders, an SPN visualization method based on this approach would allow for easy implementation in a variety of clinical situations and could be incorporated at many sites throughout the world.

The main goal of this research was to verify a proof of concept that can be developed into a solution for both illumination and acquisition. The implemented prototype has proven under clinical trial to be a comprehensive solution with high added value for basic and applied research. To the best of our knowledge, such a comprehensive product has not been presented elsewhere nor been introduced into the market so far.

### 2.1. Stabilization of Pleuroscope and Anesthesia

In the in vivo pig, the pleuroscope was stabilized using a mechanical articulating arm fixed to the operating table, minimizing hand-induced motion during filming. Short breath-holds (5–10 s) were applied to reduce respiratory motion while preserving oxygenation. For the prospective human workflow, two anesthesia strategies are envisioned: (i) local anesthesia with conscious sedation for diagnostic minipleuroscopy when only brief visualization is needed and (ii) general anesthesia with an endobronchial blocker to create working space and maximize the stability when combined with biopsy/therapy. The choice depends on clinical intent, expected procedure length, and patient comorbidities.

### 2.2. Procedure Steps and Typical Duration

**Figure 1:**
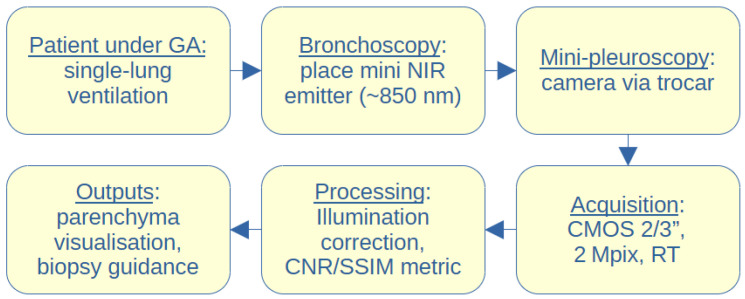
Schematic workflow of contrast-free NIR translumination for lung visualization.

Prototype workflow (see Figure 1): (1) Bronchoscopy to position the mini NIR emitter in the segmental/subsegmental bronchus; (2) a single 5–6 mm pleural port for minipleuroscopy; and (3) real-time acquisition and optional biopsy. In our preclinical setting, the individual steps were short (minutes), and the total imaging time was in the order of tens of minutes. Clinical times will vary with logistics and whether adjunctive biopsy/therapy is performed. A formal time-and-motion analysis will be included in future clinical feasibility studies.

### 2.3. Trocar Insertion and Bleeding Risk

A 5–6 mm trocar is inserted percutaneously under local anesthesia and sterile conditions, analogous to standard medical thoracoscopy. Minor soft-tissue bleeding at the skin/intercostal level may occur and is typically manageable with routine compression and local hemostasis. Pleural entry follows existing safety practices for mini-thoracoscopy; no bleeding-related complications were observed in the animal experiment.

### 2.4. Device Safety Considerations

Compared with CT (ionizing radiation), the NIR translumination device uses non-ionizing light. The emitter current was adjusted to maintain the illumination head ≤ 40 °C during prolonged operation and to avoid thermal injury; no macroscopic thermal damage was observed ex vivo or in vivo. Future work will include formal photothermal/photobiological safety testing and real-time temperature telemetry in human studies.

### 2.5. Penetration Limits and Comparison to CT

CT remains the reference for whole-thorax volumetric mapping and for detecting deeply located lesions across heterogeneous anatomy. The present NIR approach does not aim to replace CT; rather, it provides intra-thoracic, real-time visualization that can complement CT by aiding in localization during minimally invasive procedures. Ex vivo discernible parenchymal texture was observed through ∼4 cm of tissue; practical in vivo penetration depends on tissue inflation, edema, and the optical path. Further optimization (e.g., detector sensitivity, tailored wavelengths within the 800–1000 nm range, improved rejection of ambient light) may incrementally extend the effective depth, but scattering ultimately limits the performance in bulk tissue. Imaging through bone or to the intact human brain with this translumination geometry is not a target use case and is unlikely to be feasible without invasive emitter placement and rigorous safety mitigation.

### 2.6. Translational Relevance to Human Anatomy and Feasibility

Porcine lungs approximate the human parenchymal architecture but differ in lobation, chest wall thickness, and airway branching angles. Human clinical deployment will require (i) ergonomics for adult thoraces with variable BMIs; (ii) standardized calibration for scale bars; (iii) integration with standard bronchoscopic instruments and endobronchial blockers; and (iv) a clinical protocol defining indications, endpoints, and safety monitoring. A staged pathway is planned: expanded animal series → first-in-human feasibility testing (safety, image quality, CNR/SSIM) → comparative studies against navigation-assisted bronchoscopy for localization efficiency.

### 2.7. Study Design and Samples

A feasibility study with ex vivo porcine lung tissue (laboratory specimens) and a single in vivo experiment in an anesthetized, intubated pig (*n* = 1) were conducted. Ex vivo tests assessed the achievable penetration and the visibility of the fine architecture. The in vivo experiment assessed the detectability of transluminated NIR through inflated/deflated lungs from a pleural vantage point.

### 2.8. Ethics

Animal procedures were approved by the institutional ethics committee. The in vivo experiment used general anesthesia with spontaneous/assisted ventilation and unilateral mini-pleuroscopy via a 5–6 mm trocar.

### 2.9. Experimental Procedures

Ex vivo: Lung slices/blocks up to a ∼4 cm thickness were illuminated from one side, while the opposite side was imaged to assess the visibility of the lobular/septal structures and bronchial walls. In vivo: Under unilateral pneumothorax, the NIR emitter was positioned in a subsegmental bronchus under flexible bronchoscopy; a mini-pleuroscope was inserted through a single port to face the target parenchyma, and real-time video was recorded while adjusting the emitter current and camera exposure/gain.

### 2.10. Image Processing and Quantitative Metrics

Raw frames were corrected for non-uniform illumination using a median filter (3 × 3) and the subtraction of a large-kernel (101 × 101) local average, followed by optional contrast-limited adaptive histogram equalization. Quantitative metrics were defined a priori: penetration depth (max tissue thickness exhibiting discernible lobular/septal patterns), contrast-to-noise ratio (CNR) between the parenchyma structures and the adjacent background, and structural similarity (SSIM) against reference patterns (ex vivo anatomical sections or HRCT-derived textures). For the CNR, we used $\frac{\left|S_{a}-S_{b}\right|}{\sigma_{n}}$ measured from regions of interest; for SSIM, we used the standard luminance–contrast–structure formulation. All metrics were computed on temporally averaged frames to reduce shot noise.

### 2.11. Experimental Setup

The experiments were initially realized in extracted porcine lung tissues, where we tested various wavelengths in the infrared region, as well as different powers and angles of radiation. In laboratory conditions, we managed to illuminate the lung tissue with a thickness of 4 cm and could visualize the fine lung tissue architecture. Naturally, the power of the light source can be increased; however, care must be taken when heat is being released, as a change in temperature can affect the optical properties of the tissue. Therefore, we performed experiments in the range of 30–50 °C, taking into caution that a high temperature can cause local burns to the lung tissue. It is necessary to not only monitor and lower the power but also perform passive cooling, as active cooling is not possible due to size restrictions.

Once we verified the functionality of the entire solution on the extracted porcine lung tissue in the laboratory, we obtained consent from the ethical committee to perform the experiment on a pig, which will be discussed further in detail in this article.

**Figure 2:**
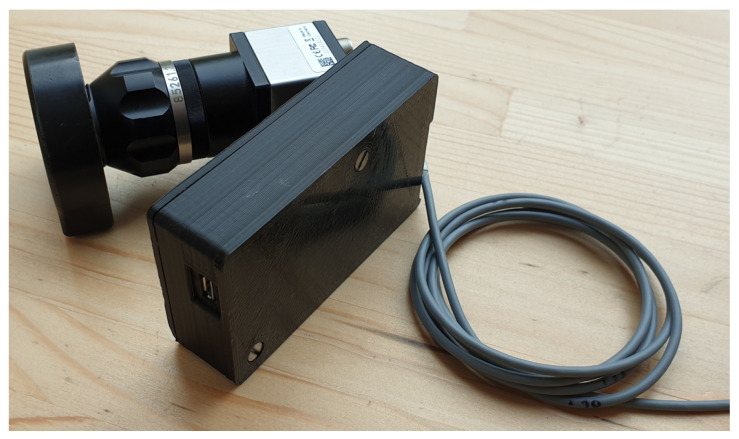
The construction of our illumination and acquisition solution.

**Figure 3:**
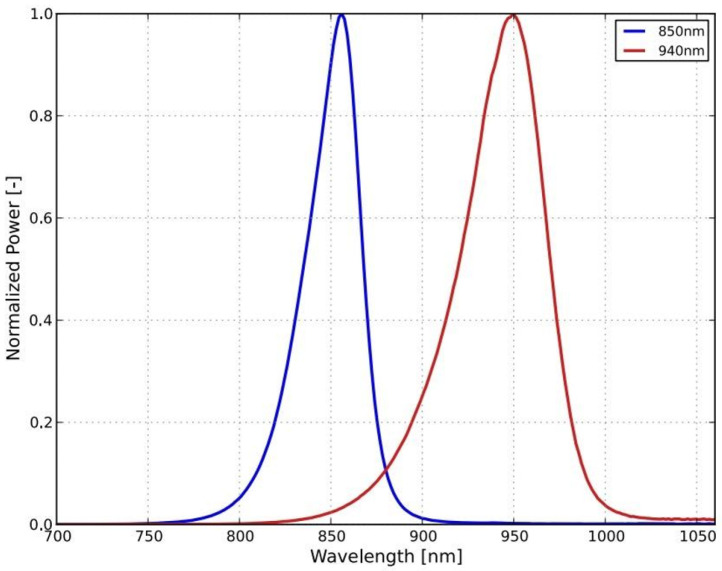
Spectral power distribution characteristics for the used LED [43] IR.

The device which we have constructed is shown in Figure 2. The in vivo part of the device consists of a lighting head and a supply cable, which is shown in Figure 2 as the gray bundle. The lighting head has an omnidirectional light source that consists of a light-emitting diode (LED) with a diffuser. It was necessary to choose a light source that was small in size and radiated only in a narrow part of the infrared spectrum. These properties were met by an electro-luminescent diode from the LUXEON IR Compact Line series (Lumileds, Eindhoven, The Netherlands), with a maximum possible radiometric power of 1050 mW and a typical spectral range from 750 nm to 950 nm, with a maximum at 850 nm (see Figure 3). The typical FWHM beam angle is 150°. The optical properties of the diode were experimentally affected by the cover layer of the nanohybrid composite, which was used to hermetically seal the lighting module. This composite enables the cooling of the LED and also works as a diffuser to obtain homogenous omnidirectional illumination around the illumination unit.

Cooling of the lighting head is ensured by passive cooling, while the heat of the LED elements is distributed to the supply cable. The measured temperature of the lighting head did not exceed 40 °C even after half an hour of use. The supply cable is led to the power source, which can be seen in Figure 2 as the black box in the middle, where there is a power supply, along with a current regulator, which enables adjustment of the light illumination intensity during sensing. Adjustment of intensity is an essential requirement for the device because to illuminate a smaller section of the lung tissue, lower exposure is required, and vice versa for a larger section. Figure 2 further shows a camera with a special mount for connection to a pleuroscope.

**Figure 4:**
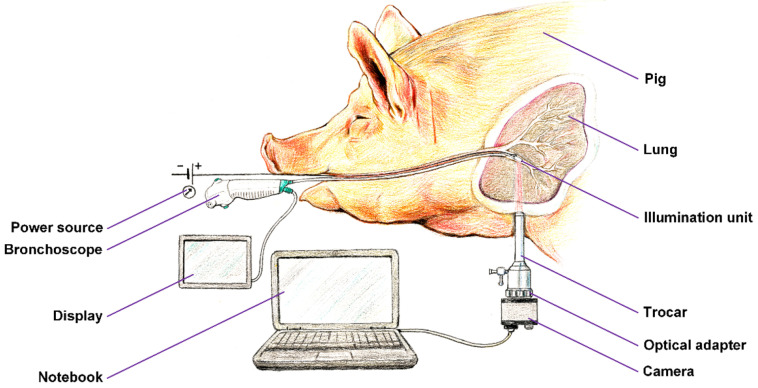
Configuration of the experiment.

We prepared a framework of the experimental setup, which is shown in Figure 4. A unilateral pneumothorax was induced in a dormant intubated pig, which was spontaneously ventilated. The source of near-infrared radiation (the illumination unit connected to a regulated power source) was introduced into its subsegmental bronchus by means of ticks and under visual inspection with a flexible bronchoscope, which allowed for either a direct view or the use of a connected display. The pig, lying on its side, was subjected to a minipleuroscopy using one port of no more than 6 mm into which the pleuroscope was inserted via a trocar. After a routine inspection of the pleural cavity, the radiation source was then emitted against the distal end of the pleuroscope, which was placed in the correct position for acquisition. Subsequently, a camera was installed on the output optics of the pleuroscope which was inserted via a trocar, and after a suitable adjustment of the power of the illumination unit, the radiation penetrating the lung tissue was detected. The real-time video stream was displayed on a notebook. After recording a high-quality video signal of the visualized tissue, the experiment was terminated.

**Figure 5:**
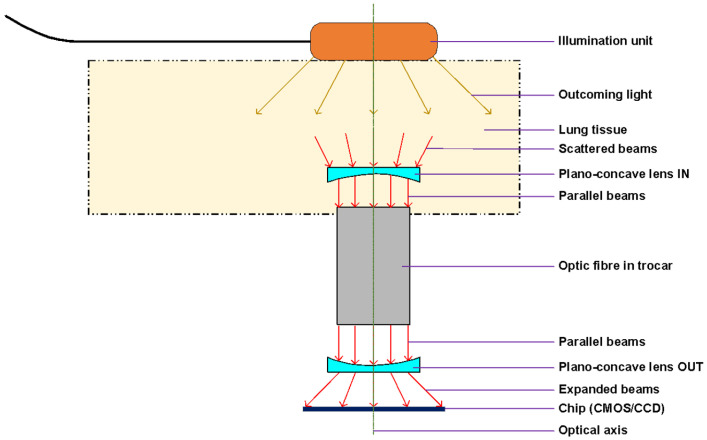
Optical principles of lung tissue translumination.

Figure 5 demonstrates the experimental setup, showing the optical principle of lung tissue translumination (*transmissive illumination*). The principle is based on the use of transmissive illumination, where the infrared light comes out from the illumination unit and is scattered in the lung tissue, which is between the light source (lighting head) and the camera. The plano-concave lens collects part of this scattered light and sends the light to the optic fiber in the trocar or the pleuroscope. For our experiment, instead of a fiber, we used the pleuroscope, which consisted of one or more optic fibers inside. From the optic fibers/pleuroscope, the parallel beams go through to the second plano-concave lens, which expands the beams to the CMOS chip, which acquires the image of the lung tissue. All optical components are centered to the optical axis of the chip.

**Figure 6:**
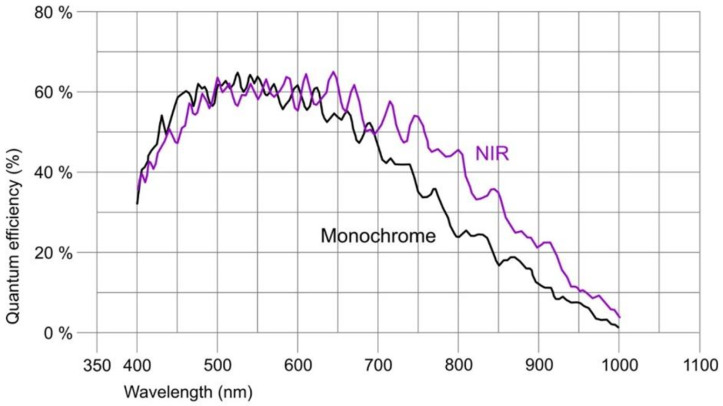
Quantum efficiency in the given wavelength range for the selected [44] imaging camera. The NIR variant was selected due to its significantly higher quantum efficiency in the 800–900 nm range.

The camera connected to a pleuroscope was equipped with a CMOS chip with a global shutter (UI-3360-CP-NIR-GL Rev.2, iDS Imaging GmbH, Obersulm, Germany). The chip of this camera is in the 2/3″ format and allows us to capture an image with a maximal size of 2048 × 1088 pixels (with a pixel size of 5.5 μm). The frame rate used was 14.78 fps. The quantum efficiency of the camera at the maximum intensity wavelength of the source is approximately 35%, which is shown in Figure 6 (the ‘NIR’ curve).

The camera was connected to the pleuroscope through a C-mount adapter (a RIWO lens with a snap-on mechanism and a C-mount thread; Richard Wolf GmbH, Knittlingen, Germany) with a focal length of *f* = 27 mm.

### 2.12. Methodology for Translumination

**Figure 7:**
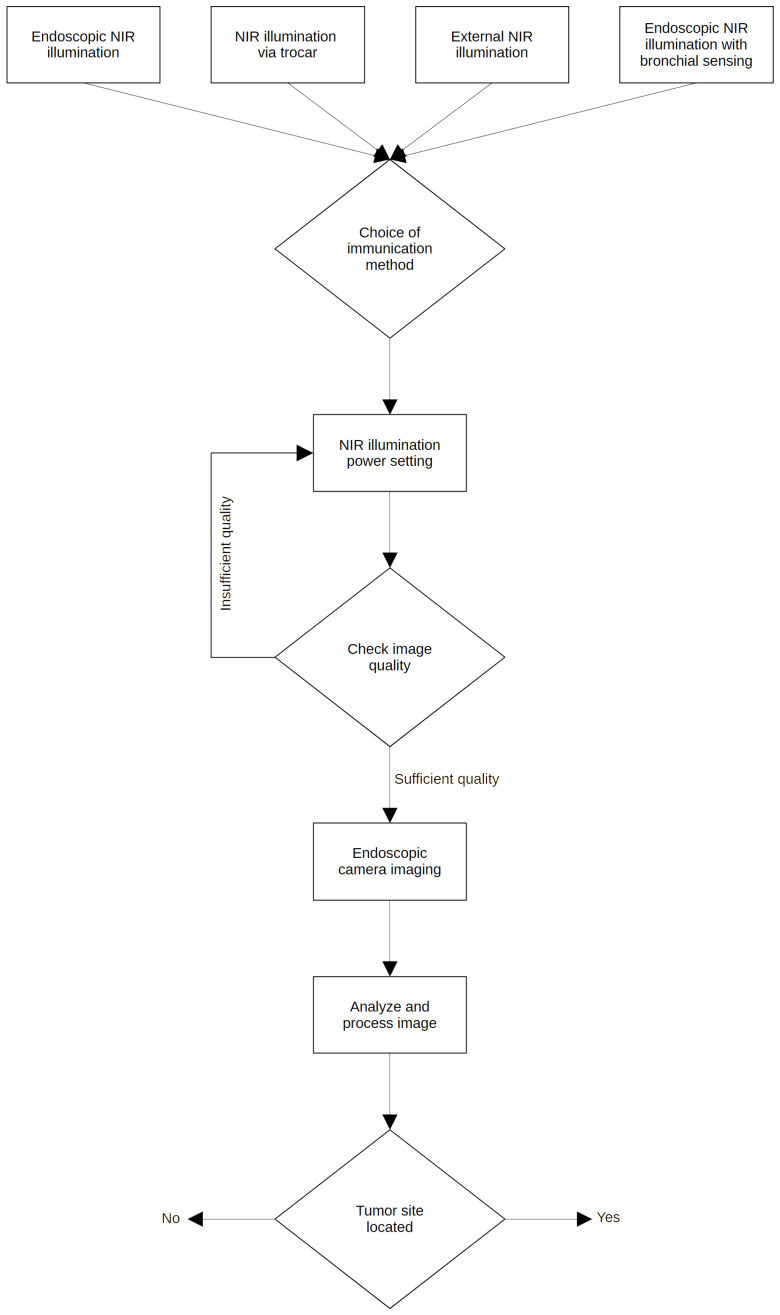
A flowchart of the whole translumination process.

We developed a methodology which is summarized in Figure 7, in the form of a flowchart, where the complete process of NIR translumination is summarized. The principle is described in detail in our utility model [45], where we protect the principle of this method and the style of use.

In all cases, the step of NIR illumination power setting follows (see Figure 7), with a default illumination power setting for each lighting method. If the image quality is inadequate, it is necessary to increase (under-illuminated tissue) or decrease (over-illuminated tissue) the power of the light unit. This can be performed either automatically by the software or by the physician, who can adjust the illumination intensity. At the same time, the acquisition parameters of the capture device, typically the camera gain and the exposure time, are also set. This is followed by the connection of the sensor unit, which in our case is the camera connected to the pleuroscope via the optical adapter. Any other type of illumination, based on experimental verification, would not fit the criteria since ambient illumination and the thickness of other tissues strongly degrade the quality of the illuminated tissue displayed. The associated software carries out analysis and image processing, for example, to highlight potential tumor foci or suspicious areas. In obvious cases, the software can evaluate the area as a pathological focus, giving the physician recommendations for findings.

## 3. Results and Discussion

Ex vivo NIR translumination revealed the alveolar-level texture and interlobular septa through 4 cm of the parenchyma under moderate emitter currents. The in vivo transluminated signal from the bronchial emitter was consistently detected from the pleural side; the lobular patterns and bronchial mucosa were visible in real time during controlled lung deflation.

Penetration depth (ex vivo): At up to a ∼4 cm tissue thickness, discernible lobular patterns were yielded at an acceptable exposure. CNR: Measurable contrast between the septa and the adjacent parenchyma was observed.

**Figure 8:**
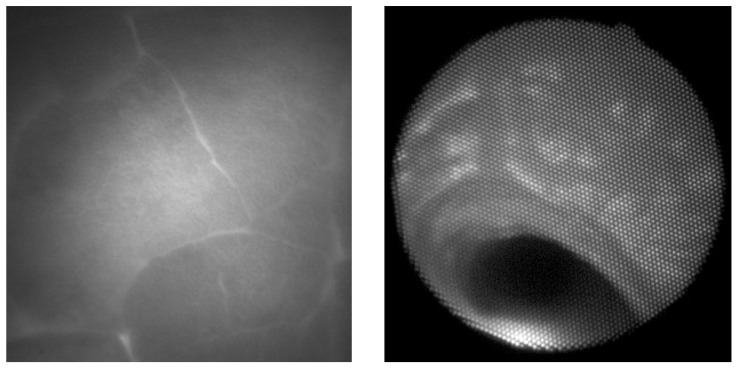
Lung tissue with a direct view (**left**); bronchial airways (multi-fiber pleuroscope) (**right**).

Two examples of acquired images are shown in Figure 8. On the left side, there is an image of a porcine lung as seen directly via the camera, and the thickness of the illuminated tissue is 4 cm. The parenchymal structure of the lung is nicely visible, including at the outline of the lobes of the lungs. On the right side, there is an image of the bronchial airway of the living pig, with illumination from the esophagus. The structure of the mucosa in the bronchial airway is also clearly visible, with the potential for further image processing and analysis.

**Figure 9:**
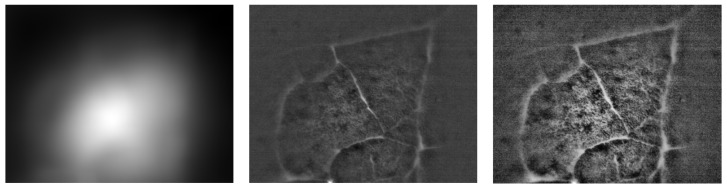
Estimation of the non-uniform illumination field (**left**), the corrected image (**middle**), and the image with adaptive histogram correction (**right**). The images are cropped in order to remove the border effect caused by image filtering. The original image is shown in Figure 8 on the left.

We performed basic image processing of the image acquired in a direct view setup. Due to the strong non-uniform illumination and level of noise in the acquired image *I*, we proposed a simple additive model for image processing. The corrected image $I_{corr}$ is computed as $I_{corr}=I_{filtered}-I*h$, where $I_{filtered}$ is the median filtered image with a 3 × 3 kernel, and *h* is a square-averaging kernel with a size of 101 pixels. An example of this processing is shown in Figure 9. The corrected image contains more visible structures, particularly the alveoli borders and the structures within. The next processing step might involve appropriate histogram adjustment. Here, we applied contrast-limited adaptive histogram equalization [46,47] in order to show the effect on the corrected image.

**Figure 10:**
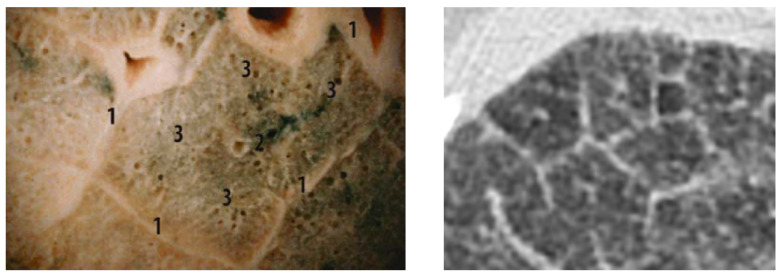
Lung lobulus anatomy: a cadaver section of the lungs with visible lung lobules with 1 for interlobular septa, 2 for central bronchioles, and 3 for alveolar region (**left**); an HRCT section of the patient with interstitial edema, where the septa and lobules are clearly visible (**right**) [48].

A comparison of the obtained images after improvements/filtering (see Figure 9) with the lung lobulus anatomy is shown in Figure 10, where the left image represents a cadaver section and the right one a *high-resolution computer tomography* (HRCT) image. It is quite obvious that we managed to achieve a completely adequate imaging method that makes the lung tissue visible in an excellent way.

The reason for our study was to answer whether the lung parenchyma could be visualized by looking from the pleural cavity in a healthy pig. The finding that this method is well suited to imaging SPNs will be the subject of further studies in the human population.

We speculated that having the possibility to place the source of NIR light into the bronchial airways, as well as simultaneously having the option to observe the pleural surface, would aid in the visualization of solitary pulmonary nodules without fluorescent dye and without the necessity of utilizing video thoracoscopy. To prove this idea, we had to confirm the possibility of the visualization of transluminated NIR light from the bronchial/esophageal area to the pleural cavity or/and trachea. Having such an option would lead to a substantial improvement in the diagnostic approach to solitary pulmonary nodules, as well as a decrease in diagnostic costs. The possibility of direct visualization of solitary lung lesions through translumination with different light sources has not been described or investigated in the area of the lung parenchyma. The indisputable advantage of such an approach potentially includes the ability for routine clinical video/pleuroscopy with the possibility of real-time tissue biopsy under direct visual control and even SPN treatment in one session. As mentioned before, since clinical pleuroscopy is an examination that has practically no contraindications, a method that allows for the visualization of solitary pulmonary nodes would be very easy to apply to a variety of clinical situations, as well as incorporate at many sites throughout the world.

Technically, no issues arise when placing the NIR light source into the distal bronchial airways during bronchoscopy under general anesthesia. The only issue would be to create sufficient space for a diagnostic visualization device (minipleuroscope) that would be introduced and adequately targeted in the pleural cavity. In order to solve this, 3 years ago, we developed and repeatedly used an endobronchial blocker technique in patients undergoing clinical pleuroscopy. During the procedure, we introduced a blocked device into the main bronchus on the examined site. After introducing a mini-trocar (5 mm) into the pleural cavity, we gently deflate the lung by channeling in the blocker device and introducing a minipleuroscope into the pleural cavity. Hence, we decided there was no principle-related nor technical obstacle to organizing the animal experiment.

This feasibility work demonstrates that dye-free NIR translumination can be used to visualize the lung parenchyma from a pleural vantage by placing an internal emitter in the airway. This complements the ICG-based fluorescence techniques used for thoracic surgery by eliminating systemic dye exposure and simplifying the logistics, at the expense of relying on intrinsic optical contrast. Key limitations include the small sample size (the ex vivo laboratory specimens; a single in vivo pig), the absence of human data, a lack of absolute spatial calibration in the current setup (precluding scale bars in some images), and the preliminary status of the quantitative validation. Future work will expand the number of animals, standardize the calibration to enable scale bars on all figures, and report the per-case CNR, gCNR, and SSIM against an anatomical reference. Clinical translation will focus on integrating the emitter into standard bronchoscopic tooling and on the mini-pleuroscopy workflows for combined visualization and biopsy.

In porcine tissue and a single in vivo pig, contrast-free NIR translumination enabled real-time visualization of the lung parenchyma from the pleural side with an airway-placed emitter. These results establish a proof of feasibility only; additional quantitative validation and human studies are required before any claims on diagnostic performance or clinical outcomes can be made.

## Data Availability

The data presented in this study are available on request from the corresponding author. The data are not publicly available due to still running own research activity.

## Abbreviations

The following abbreviations are used in this manuscript:

AFB	Autofluorescence Bronchoscopy
CCD	Charge-Coupled Device
CMOS	Complementary Metal-Oxide-Semiconductor
CT	Computer Tomography
DALY	Disability-Adjusted Life-Year
FEV1	Forced Expiratory Volume in 1 Second
FWHM	Full Width at Half Maximum
HRCT	High-Resolution Computer Tomography
LED	Light-Emitting Diode
NIR	Near-Infrared
NLST	National Lung Screening Trial
RIWO	Reduced I/O Working
QUALY	Quality-Adjusted Life-Year
SPN	Solitary Pulmonary Nodule
WLF	White Light Bronchoscopy
